# Mitochondrial dysfunction and immune suppression in BRAF V600E‐mutated metastatic melanoma

**DOI:** 10.1002/ctm2.1773

**Published:** 2024-07-19

**Authors:** Natália Pinto de Almeida, Ágnes Judit Jánosi, Runyu Hong, Ahmad Rajeh, Fábio Nogueira, Leticia Szadai, Beata Szeitz, Indira Pla Parada, Viktória Doma, Nicole Woldmar, Jéssica Guedes, Zsuzsanna Újfaludi, Aron Bartha, Yonghyo Kim, Charlotte Welinder, Bo Baldetorp, Lajos Vince Kemény, Zoltan Pahi, Guihong Wan, Nga Nguyen, Tibor Pankotai, Balázs Győrffy, Krzysztof Pawłowski, Peter Horvatovich, Attila Marcell Szasz, Aniel Sanchez, Magdalena Kuras, Jimmy Rodriguez Murillo, Lazaro Betancourt, Gilberto B. Domont, Yevgeniy R. Semenov, Kun‐Hsing Yu, Ho Jeong Kwon, István Balázs Németh, David Fenyő, Elisabet Wieslander, György Marko‐Varga, Jeovanis Gil

**Affiliations:** ^1^ Laboratory of Proteomics/LADETEC Universidade Federal Do Rio de Janeiro Rio de Janeiro Brazil; ^2^ Proteomics Unit Institute of Chemistry Universidade Federal Do Rio de Janeiro Rio de Janeiro Brazil; ^3^ Clinical Protein Science & Imaging, Biomedical Centre Department of Biomedical Engineering Lund University Lund Sweden; ^4^ Department of Dermatology and Allergology University of Szeged Szeged Hungary; ^5^ Institute for Systems Genetics NYU Grossman School of Medicine New York City New York USA; ^6^ Department of Biochemistry and Molecular Pharmacology NYU Grossman School of Medicine New York City New York USA; ^7^ Department of Dermatology Massachusetts General Hospital Harvard Medical School Boston Massachusetts USA; ^8^ Department of Internal Medicine and Oncology Semmelweis University Budapest Hungary; ^9^ Section for Clinical Chemistry Department of Translational Medicine Lund University Lund Sweden; ^10^ 2nd Department of Pathology Semmelweis University Budapest Hungary; ^11^ Department of Dermatology Venerology and Dermatooncology Semmelweis University Budapest Hungary; ^12^ Department of Pathology Albert Szent‐Györgyi Medical School University of Szeged Szeged Hungary; ^13^ Competence Centre of the Life Sciences Cluster of the Centre of Excellence for Interdisciplinary Research Development and Innovation University of Szeged Szeged Hungary; ^14^ Department of Bioinformatics Semmelweis University Budapest Hungary; ^15^ Data Convergence Drug Research Center Therapeutics and Biotechnology Division Korea Research Institute of Chemical Technology (KRICT) Daejeon South Korea; ^16^ Division of Oncology Department of Clinical Sciences Lund Lund University Lund Sweden; ^17^ HCEMM‐SU Translational Dermatology Research Group Semmelweis University Budapest Hungary; ^18^ Department of Physiology Semmelweis University Budapest Hungary; ^19^ Department of Biomedical Informatics Harvard Medical School Boston Massachusetts USA; ^20^ Genome Integrity and DNA Repair Core Group Hungarian Centre of Excellence for Molecular Medicine (HCEMM) University of Szeged Szeged Hungary; ^21^ Cancer Biomarker Research Group Research Centre for Natural Sciences Budapest Hungary; ^22^ Department of Molecular Biology University of Texas Southwestern Medical Center Dallas Texas USA; ^23^ Department of Biochemistry and Microbiology Warsaw University of Life Sciences Warsaw Poland; ^24^ Department of Analytical Biochemistry Faculty of Science and Engineering University of Groningen Groningen The Netherlands; ^25^ Chemical Genomics Global Research Lab Department of Biotechnology College of Life Science and Biotechnology Yonsei University Seoul Republic of Korea; ^26^ 1st Department of Surgery Tokyo Medical University Tokyo Japan; ^27^ Department of Biophysics, Medical School University of Pecs Pecs Hungary

Dear Editor,

Our investigation into the mitochondrial proteome dynamics in metastatic melanoma reveals profound metabolic reprogramming that is central to the progression and treatment resistance of BRAF V600E‐mutated tumours. These findings highlight significant alterations in mitochondrial function and immune response, opening new avenues for precision medicine by identifying targeted treatments that could disrupt these adaptive pathways and lead to more effective therapeutic strategies. Melanoma, characterized by its aggressive behaviour and high metastatic capacity, faces further challenges with the BRAF V600E mutation, which significantly drives cancer progression and impacts treatment responses.^1^ Building upon our previous insights into melanoma's molecular dynamics, this study delves deeper into mitochondrial proteome alterations associated with the BRAF V600E mutation.^2^, ^3^ Through comprehensive analysis of proteomic data from a total of 127 melanoma lymph node metastases, sourced from the Human Melanoma Proteome Atlas project, we demonstrate how specific mitochondrial disruptions enhance melanoma proliferation and facilitate progression towards distant metastasis (Figure 1A).^4^ By investigating the specific role of mitochondrial dysfunction in the progression and immune evasion of BRAF V600E‐mutated metastatic melanoma, we aim to elucidate the molecular mechanisms that drive aggressive melanoma behaviour and identify potential targets for precision medicine. This approach highlights the critical interplay between mitochondrial function and immune response, paving the way for innovative therapeutic strategies to combat this challenging form of cancer.

**FIGURE 1:**
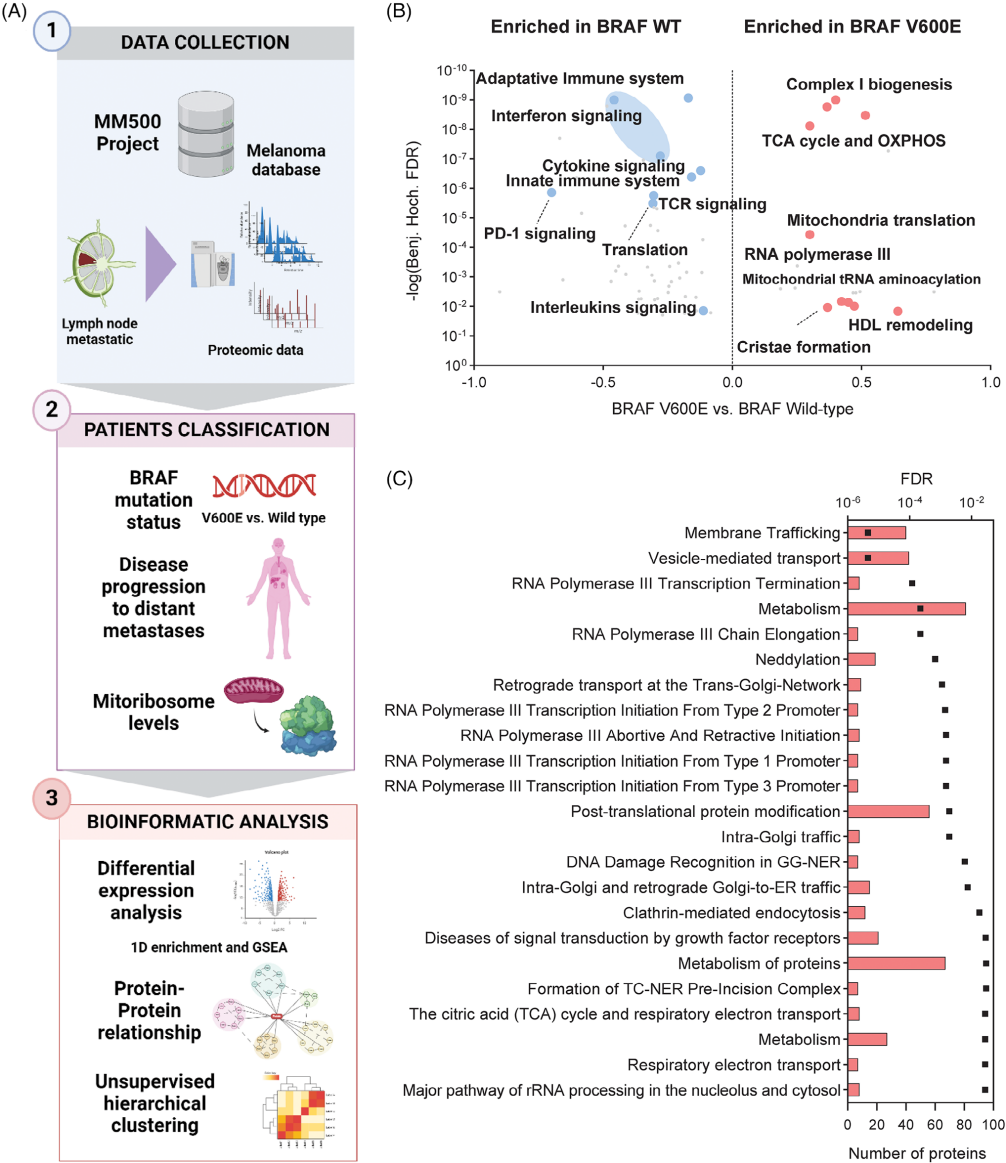
Overview of study design for investigating mitochondrial proteome dynamics in melanoma metastases. (A) Schematic representation of the workflow and objectives of our research, which utilizes data from lymph node metastases within the Human Melanoma Proteome Atlas project. We conducted an extensive bioinformatic analysis to explore the interconnections between BRAF mutation status, disease progression and alterations in the mitochondrial proteome, to uncover specific mitochondrial signatures that correlate with disease aggressiveness, providing insights into targeted therapeutic strategies. (B) Volcano plot representation of the results of the 1D functional annotation enrichment analysis (Perseus 1.6.15.0) based on REACTOME pathways, showing proteomic differences between BRAF V600E and wild‐type (WT) lymph node tumours in Cohort 2 (FDR < .02). Analysis of Cohort 1 is detailed in Figure S1. (C) Functional enrichment analysis of significantly upregulated proteins in the mutated tumours. STRING platform (https://string‐db.org) (Last accessed: 2023‐05‐04).

In this study, we utilized proteomic data from 127 melanoma metastasis samples. Out of these, 78 samples included information about the BRAF mutation status, with 45 harbouring the BRAF V600E mutation and 33 displaying the wild‐type (WT) variant. The WT group does not have GTPase NRas (NRAS) mutations; however, the Neurofibromin (NF1) mutation status is not known for these samples. To investigate the molecular signatures linked to melanoma progression towards distant metastasis, we analysed proteomic data from 88 lymph node metastases with known progression status. These were divided into two groups: patients who developed distant metastases (*n* = 54) and patients without progression (*n* = 34). Due to the observed significance of mitochondrial translational machinery in both BRAF mutation status and disease progression, we focused on mitochondrial ribosome proteins (MRPs). For this analysis, we selected data from 88 metastasis samples with a tumour content higher than 50% for hierarchical clustering and heatmap visualization.

The BRAF V600E mutation, prevalent in over 50% of melanoma cases, significantly drives cancer progression by activating metabolic and proliferative pathways.^5^ Our proteomic analysis comparing BRAF V600E mutated with wild‐type samples highlights stark differences in mitochondrial function and immune responses. Key mitochondrial processes such as the tricarboxylic acid (TCA) cycle, oxidative phosphorylation (OXPHOS) and mitochondrial translation machinery are significantly enhanced in BRAF V600E samples. Furthermore, upregulation of RNA polymerase III activity, linked to tumour progression, and downregulation of immune response pathways, including PD‐1 signalling, suggest mechanisms through which these tumours may evade immune detection, emphasizing mitochondrial dysfunction and immune suppression as potential therapeutic targets (Figure 1B).

Our functional enrichment analysis of significantly dysregulated proteins reveals heightened mitochondrial functions in BRAF V600E‐mutated melanoma metastases. Notably upregulated proteins involved in OXPHOS, RNA polymerase III transcription and mitochondrial translation support a metabolic adaptation geared towards increased energy production and rapid cell proliferation. Proteins critical for maintaining mitochondrial integrity, aiding in mitochondrial transport, protein import and cristae formation are also enriched, reinforcing the robust mitochondrial functionality necessary for the metabolic reprogramming of melanoma cells (Figure 1C).

Further detailed analysis of mitochondrial translation and OXPHOS proteins validates our findings. Significant upregulation of key proteins critical for mitochondrial energy production, such as those involved in tRNA aminoacylation and rRNA methylation, along with components crucial for OXPHOS, particularly in Complex I, supports this metabolic stage (Figure 2A,C). Transcriptomic data from the The Cancer Genome Atlas Program (TCGA) corroborate these proteomic findings, showing upregulation of OXPHOS‐related genes in BRAF‐mutated samples, underlining the link between mitochondrial functionality and the aggressive nature of these tumours (Tables S4 and S5).

**FIGURE 2:**
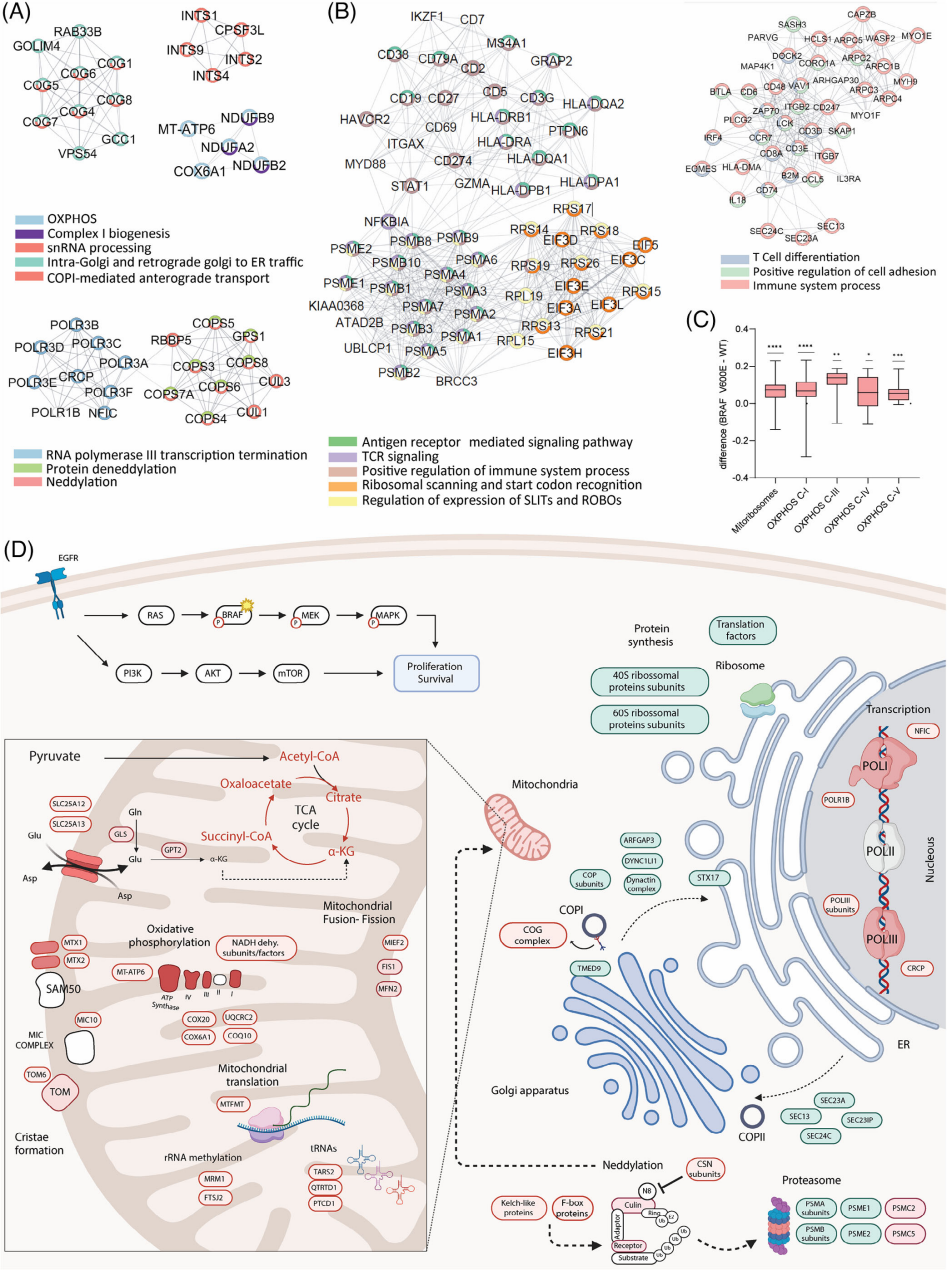
Comprehensive analysis of pathway dysregulation in BRAF V600E‐mutated lymph node metastases. (A) Significant clusters of proteins upregulated in the BRAF‐mutated tumours identified through MCODE (v.2.0.2) analysis of STRING (v.2.0.1) networks (Cytoscape v.3.9.1). (B) Significant clusters of proteins downregulated in the BRAF‐mutated tumours identified by MCODE analysis of STRING networks. (C) Relative expression levels of mitochondrial ribosome and oxidative phosphorylation (OXPHOS) proteins across samples, with statistical significance marked by asterisks where *, **, *** and **** correspond to *p*‐values < .05, .01,    .001 and .0001, respectively, determined by a one‐sample *t*‐test. (D) Schematic representation of the pathways dysregulated in BRAF V600E metastatic lymph nodes. This panel graphically depicts the upregulated proteins in the BRAF‐mutated tumours in red, and the downregulated proteins in green.

Moreover, mitochondrial dynamics and biogenesis are enhanced in BRAF V600E‐mutated metastatic melanomas, evidenced by the upregulation of proteins like Metaxin‐1 and 2 (MTX1/2) and Mitochondrial import receptor subunit TOM6 homolog (TOMM6) and corroborated by transcriptomics that show genes associated with these functions are linked to increased survival, proliferation and metastatic potential by altering energy production and cellular redox states.^2^, ^6^ Concurrently, glutamine metabolism, crucial in BRAF‐mutated melanoma, involves upregulated mitochondrial transporters that enhance glutamine conversion into essential metabolites for proliferation (Figure S2). Additionally, the neddylation pathway, which regulates protein ubiquitination and degradation,^7^ enhances mitochondrial functionality and regulatory control over the cell cycle and immune responses, indicating the metabolic reprogramming characteristic of this aggressive cancer (Figure 2D).

Conversely, BRAF‐mutated melanoma metastases exhibit disruptions in intracellular transport, notably affecting the Golgi apparatus and endoplasmic reticulum, which impact crucial cellular operations and the interaction between mitochondria and cellular dynamics. Critically, there is substantial downregulation of immune system response pathways, including components like T cell activation and Human leukocyte antigen (HLA) class II molecules, likely contributing to immune evasion, enhancing tumour progression, and resistance to treatments (Figure 2B). These findings underscore the complex challenge in effectively targeting BRAF‐mutated melanoma and highlight the intricate interplay between mitochondrial metabolism and immune evasion in this context.

Next, we explored the critical roles of mitochondrial dysregulation and immune suppression in driving melanoma progression towards distant metastasis by analysing proteomic data from 88 lymph node metastases. This included a distinction between those associated with distant metastases and those without progression (Table S1). Histological assessments showed no significant differences in tumour cell and connective tissue content between the groups; however, a marked reduction in lymphocyte distribution in the progression group suggests a potential immune evasion mechanism (Figure S4). Functional enrichment analyses highlighted that mitochondrial pathways, particularly mitochondrial translation, are closely associated with the development of distant metastases regardless of the BRAF mutation status. Proteins involved in cristae formation, pyruvate metabolism, the TCA cycle and OXPHOS were significantly upregulated in the progression group of BRAF‐mutated samples, underscoring their importance in melanoma's metabolic regulation and progression (Figures 3A−D; Tables S6−S8). Transcriptomic data from the TCGA database further supported the pivotal role of these mitochondrial pathways in influencing melanoma‐specific survival, with multivariate Cox analysis revealing complex correlations with survival outcomes in advanced‐stage melanoma patients (Table S2).

**FIGURE 3:**
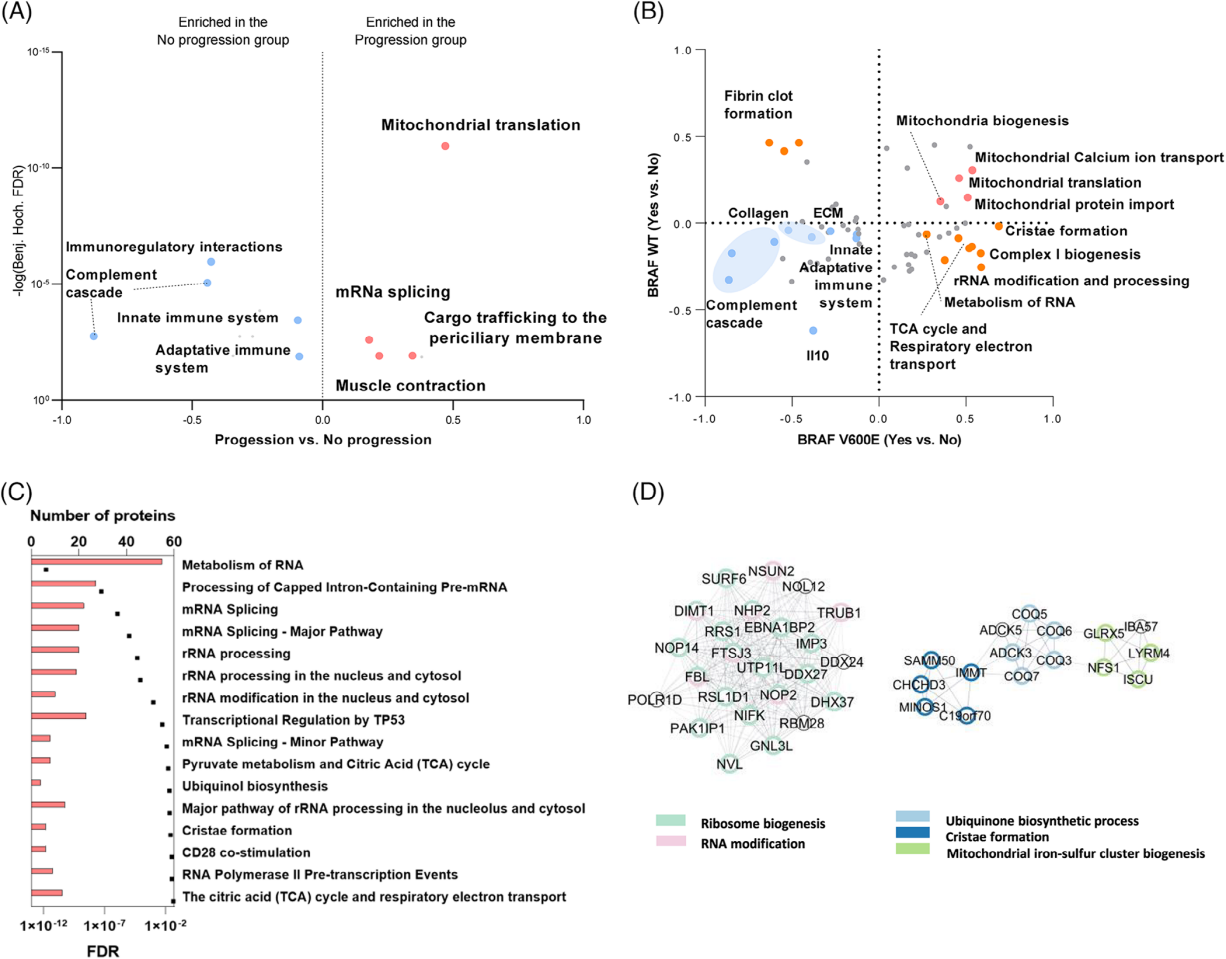
The impact of mitochondrial activation and immune system downregulation on the development of distant metastasis. (A) The panel displays the results of 1D functional annotation enrichment analysis (Perseus 1.6.15.0) for comparing proteome dynamics between progression and no progression groups (FDR < .02). Reactome pathways were used for the functional annotation of the proteins. Pathways upregulated in patients who developed distant metastases are in red, and downregulated pathways are in blue. (B) 2D enrichment functional annotation enrichment analysis results related to the progression analysis in both BRAF mutated and WT samples. Reactome pathways upregulated in patients developing distant metastases across both BRAF mutated and WT groups are shown in red, while downregulated pathways are shown in blue. Pathways that are differentially dysregulated during progression in BRAF mutated versus WT tumours are highlighted in orange (FDR < .02). (C) Functional enrichment analysis of proteins upregulated in the progression group carrying the BRAF mutation. STRING platform (https://string‐db.org) (Last accessed: 2023‐02‐05). (D) Significant clusters of proteins upregulated in the progression group identified by MCODE analysis (v.2.0.2) (Cytoscape v.3.9.1).

To gain insights into the molecular mechanisms underlying mitochondrial translation in melanoma, we conducted bioinformatics analyses focusing on the MRPs. Our analysis indicates that high levels of MRPs are associated with enhanced OXPHOS and extensive mitochondrial metabolic activities, including DNA repair mechanisms and intra‐Golgi protein trafficking. This underscores the role of mitochondrial translation components in driving melanoma progression by modulating key cellular functions. Such robust mitochondrial activity may compromise immune surveillance, thereby facilitating tumour evasion (Figures 4A−C; Tables S9−S11). Conversely, tumours with lower mitoribosome levels were enriched for proteins involved in extracellular matrix organization, including collagen metabolism (Figure 4B), indicating a potential link between mitochondrial function and extracellular matrix dynamics.^8^ Mitoribosome levels negatively correlate with immune system responsiveness, particularly with processes related to the innate immune system, such as the complement cascade (Figure 4C). This association suggests that robust mitochondrial activity in melanoma could foster mechanisms that suppress immune surveillance and promote tumour evasion, consistent with findings that link dysregulated mitochondrial translation to impaired immune responses, including altered antigen presentation and immune cell exhaustion.^9^

**FIGURE 4:**
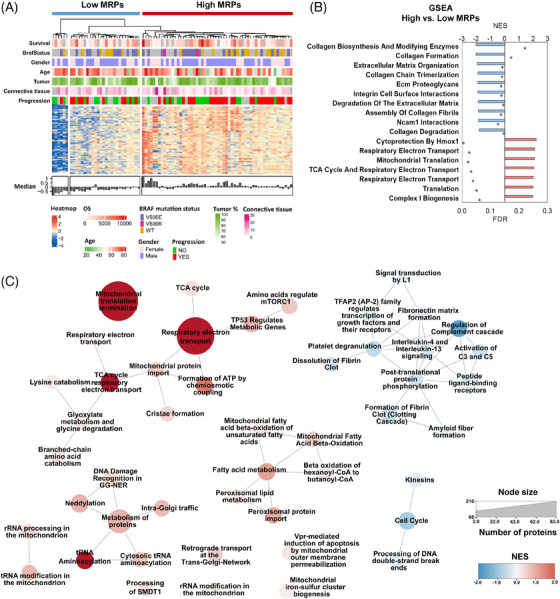
Clustering and pathway analysis based on mitochondrial ribosome proteins (MRPs) in melanoma metastasis. (A) Unsupervised hierarchical clustering showing patient stratification based on MRP levels, employing Euclidean distance and Ward's method for clustering. R v4.2.0. RStudio 2022.07.2‐576. Package ComplexHeatmap version 2.18.0. (B) Gene Set Enrichment Analysis (GSEA; v.4.3.2) comparing patients with high versus low levels of MRPs. Red bars indicate reactome pathways significantly upregulated in patients with higher MRP levels, and blue bars represent pathways downregulated (FDR < .25). (C) An Enrichment Map (v.3.3.6) (Cytoscape 3.9.1) constructed from the results of functional enrichment analysis based on protein clusters significantly correlated with MRP levels, as determined by Pearson correlation analysis (adjusted *p*‐value < .05) (R v.4.2.0. RStudio 2022.07.2‐576). Blue nodes depict pathways that are downregulated, while red nodes indicate upregulated pathways. Each node's size corresponds to the Normalized Enrichment Score (NES), providing a visual representation of the impact of MRP levels on metabolic and regulatory pathways in melanoma metastases.

While our study offers valuable insights into the mitochondrial proteome dynamics and immune suppression in BRAF V600E‐mutated metastatic melanoma, there are several limitations to acknowledge. Firstly, the relatively small sample size may limit the generalizability of our findings. Secondly, our analysis is predominantly based on bioinformatics predictions and proteomic data, which, although robust, require further functional validation in biological models. Addressing these limitations in future research will be crucial for confirming our findings and translating them into clinical applications. Despite these limitations, our study lays a strong foundation for understanding the complex interplay between mitochondrial dysfunction and immune suppression in metastatic melanoma, paving the way for targeted therapeutic strategies.

Our findings underscore the potential of targeting mitochondrial functions with precision medicine, such as using mitochondrial translation inhibitors like tetracyclines and macrolides, to curb the metabolic flexibility vital for melanoma progression. The ongoing clinical trial (NCT03026517) tests a combination of Phenformin, a complex I inhibitor, with BRAF and MEK inhibitors to exploit the metabolic vulnerabilities of BRAF‐mutated melanomas.^10^ Furthermore, our results support the combination of mitochondrial targeting with immune checkpoint inhibitors, effectively countering both tumour growth and immune evasion. This approach paves the way for innovative combination therapies that concurrently tackle metabolic reprogramming and immune suppression in melanoma. Additionally, our study highlights the importance of integrating metabolic and immune‐targeting strategies to enhance therapeutic efficacy. By identifying the interplay between mitochondrial dysfunction and immune suppression, we provide a rationale for combining therapies that inhibit mitochondrial translation and metabolic pathways with those that activate the immune response. This combination could potentially overcome the adaptive resistance mechanisms of melanoma cells, leading to more durable treatment responses. Future research should focus on preclinical and clinical studies to evaluate the efficacy of these combination therapies, explore optimal dosing regimens and identify biomarkers for patient stratification and response monitoring. Ultimately, these efforts could lead to the development of personalized treatment strategies that improve outcomes for patients with BRAF V600E‐mutated metastatic melanoma.

## AUTHOR CONTRIBUTIONS

Study concept: G.M.‐V. and J.G.; Methodology: N.P.A., A.J.J., R.H., A.R., I.P.P., N.W., J. Guedes., and J.G.; Data collection and analysis: N.P.A., A.J.J, L.S., N.W., J. Guedes., Y.K., C.W., Z.P., G.W., N.N., A.M.S., A.S., M.K., J.R.M., L.B., and J.G.; Supervision: F.N., Z.U., B.B., L.V.K., T.P., B.G., K.P., P.H., G.B.D., Y.R.S., K.‐H.Y., H.J.K., I.B.N., D.F., E.W., G.M.‐V., and J.G.; Writing original draft: N.P.A. N.W., J. Guedes., G.M.‐V., and J.G.; Writing final version: N.P.A. N.W., J. Guedes., G.M.‐V., and J.G. Review andediting: all authors; Scientific oversight of the study: all authors.

## CONFLICT OF INTEREST STATEMENT

The authors declare no potential conflict of interest.

## ETHICS STATEMENT

The project and the study workflow were approved by the local ethical committees; at Lund University, Southern Sweden (DNR191/2007, BioMEL biobank 101/2013, 2015/266 and University of Szeged, Hungary (MEL‐PROTEO‐001). The study was conducted following relevant guidelines and regulations from the Swedish biobanking laws and Declarations of Helsinki.

## Supporting information

Supporting Information

Supporting Information

Supporting Information

Supporting Information

Supporting Information

Supporting Information

Supporting Information

Supporting Information

Supporting Information

Supporting Information

Supporting Information

Supporting Information

Supporting Information

Supporting Information
