# Supplementary material for: Mitochondrial dysfunction and immune suppression in BRAF V600E‐mutated metastatic melanoma
Source: Clin Transl Med. 2024 Jul 19;14(7):e1773. doi: 10.1002/ctm2.1773 (PMC11259597; doi:10.1002/ctm2.1773)
Supplement: Supplementary file 1 — Supporting Information [file CTM2-14-e1773-s007.docx]

**Targeting mitochondrial dysfunction and immune suppression in BRAF V600E mutated metastatic melanoma**

**Supplemental figures**

**
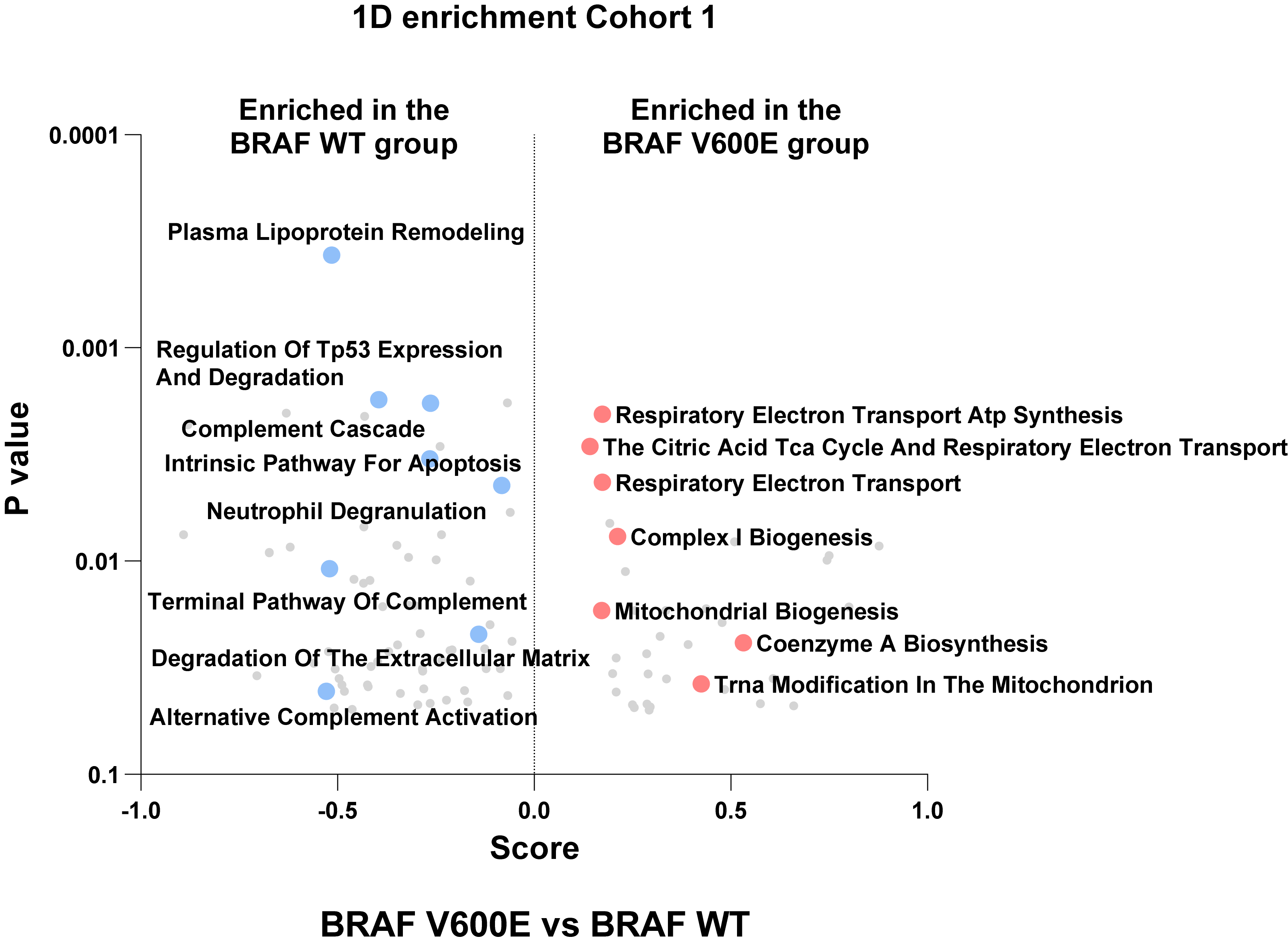
**

**Figure S1: 1D functional annotation enrichment analysis of the proteome dynamics between BRAF mutation status groups in cohort study 1.** Results from cohort 2 are presented in the main manuscript.

**
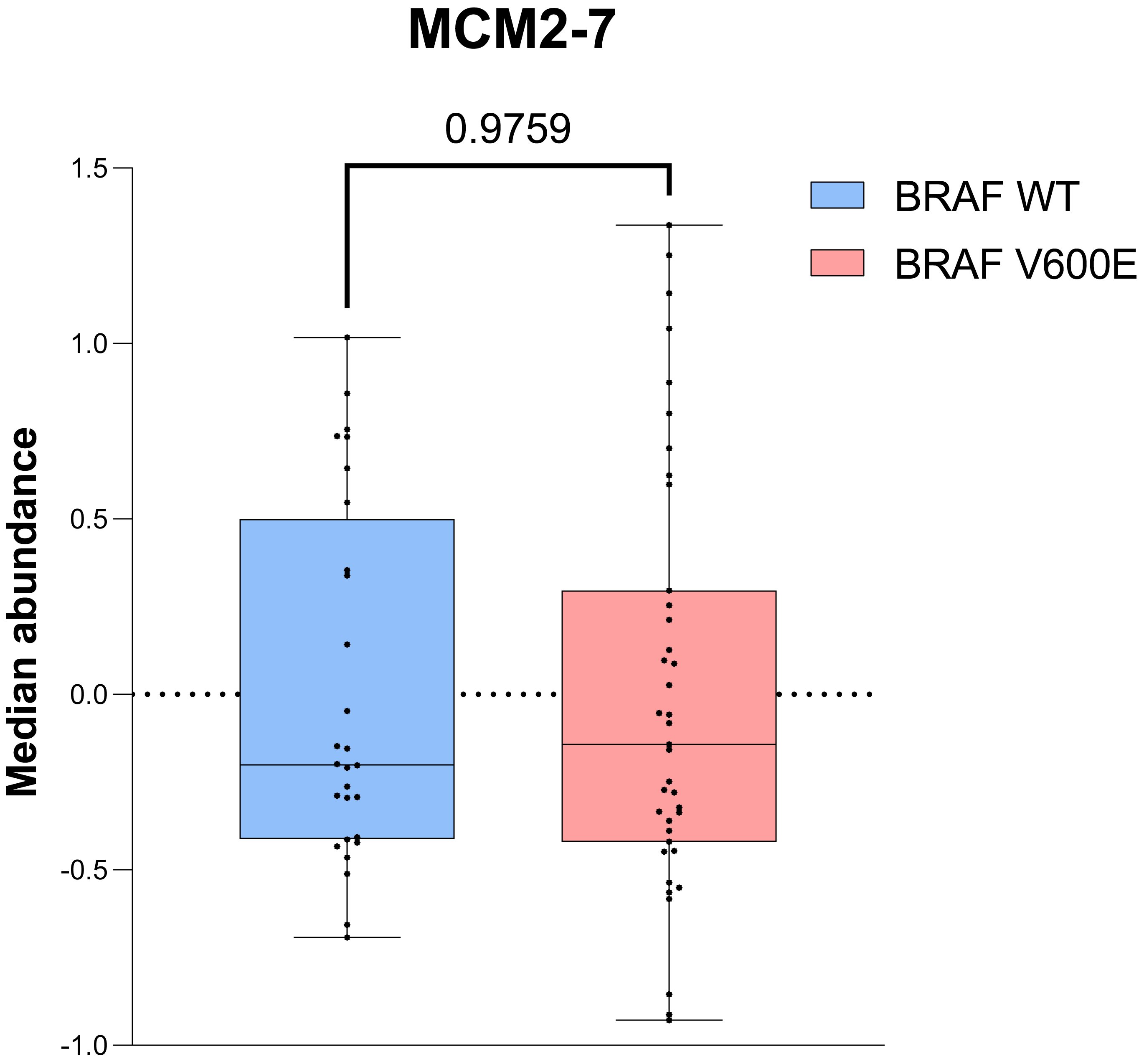
**

**Figure S2: Comparison of the median abundance of MCM complex proteins 2-7 between tumors showing BRAF wild type and mutated V600E.** The comparative analysis of the proliferation status between BRAF mutation groups, using the mini-chromosome maintenance (MCM) complex as a proxy [43–46].

**
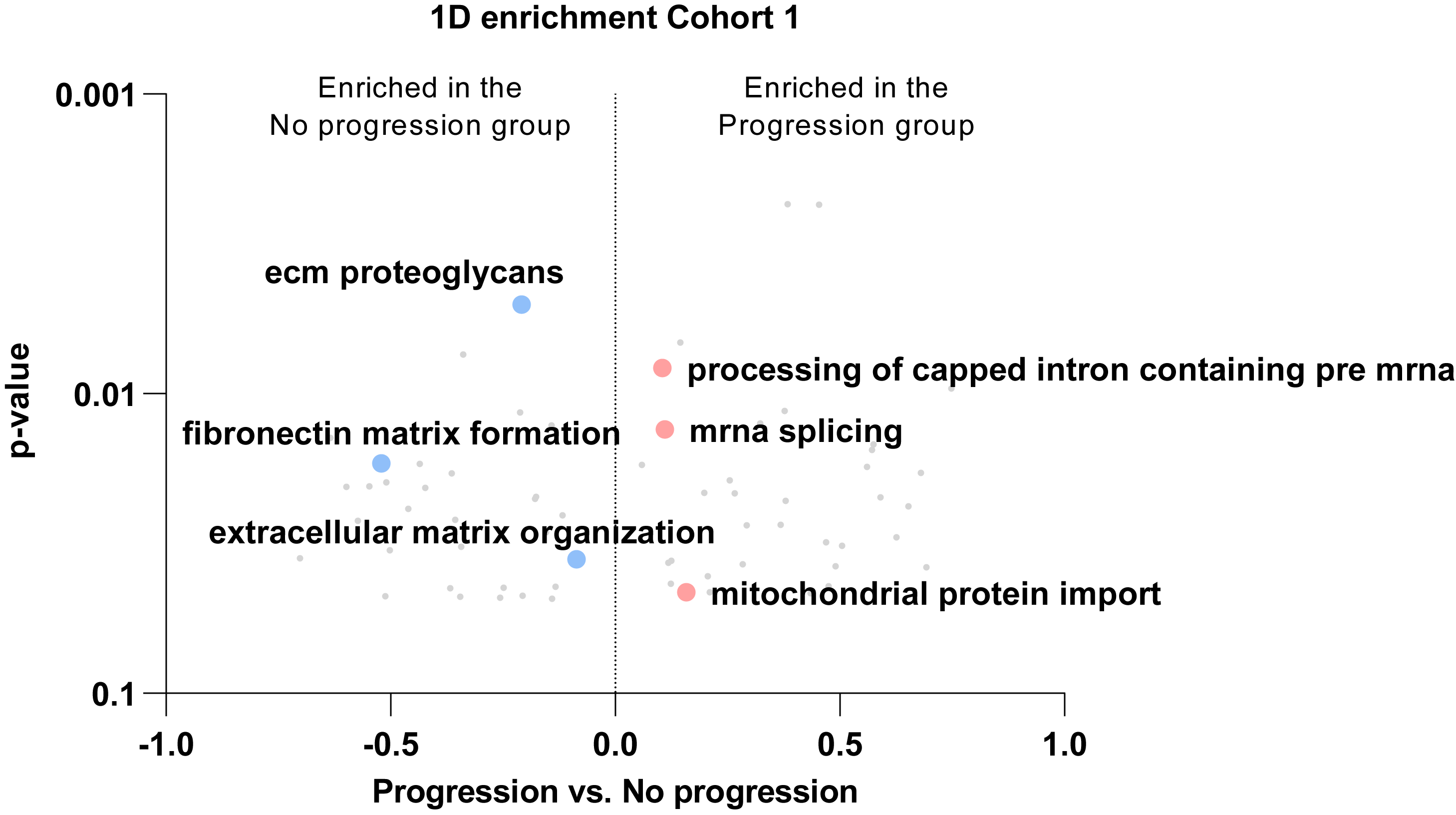
**

**Figure S3: 1D functional annotation enrichment analysis of the proteome dynamics between metastasis groups based on the progression towards distant metastases in the cohort study 1.** Results from cohort 2 are presented in the main manuscript.

**
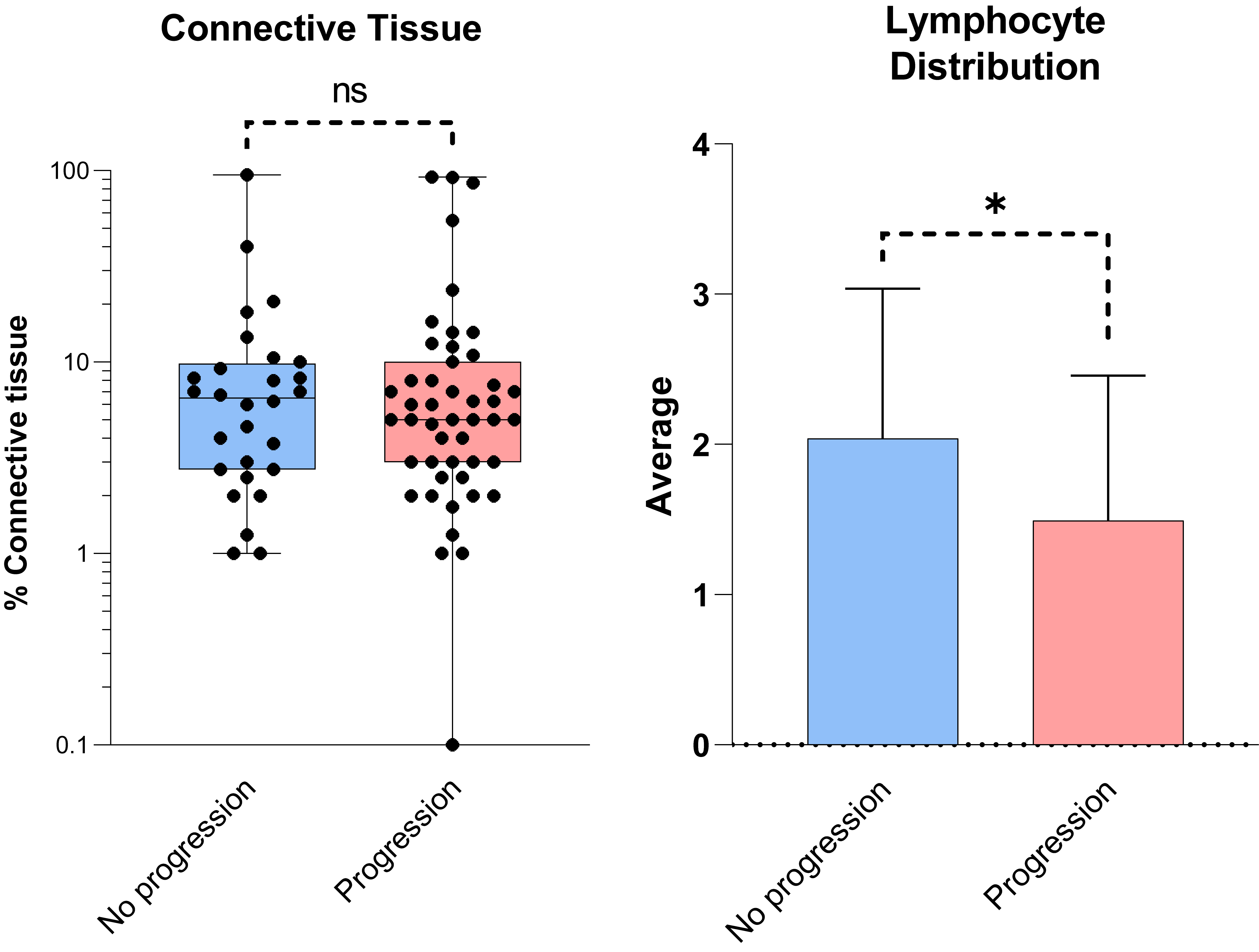
**

**Figure S4: Evaluation of connective tissue content and lymphocyte distribution in the progression groups.** Unpaired Mann-Whitney test analysis. (*) p-value<0.05.
