# Supplementary material for: Mitochondrial dysfunction and immune suppression in BRAF V600E‐mutated metastatic melanoma
Source: Clin Transl Med. 2024 Jul 19;14(7):e1773. doi: 10.1002/ctm2.1773 (PMC11259597; doi:10.1002/ctm2.1773)
Supplement: Supplementary file 2 — Supporting Information [file CTM2-14-e1773-s009.docx]

**Targeting mitochondrial dysfunction and immune suppression in BRAF V600E mutated metastatic melanoma**

**Supplemental information**

**Methods**

***Clinical data***

The Proteomics data analyzed in this study was obtained from the cohorts described in “The Human Melanoma Proteome Atlas[1–3]. The project was approved by the local ethical committees; at Lund University, Southern Sweden (DNR 191/2007, BioMEL biobank 101/2013, 2015/266 and 2015/618), at Semmelweis University, Hungary (191-4/2014), and University of Szeged, Hungary (MEL-PROTEO-001). Data were selected from the analysis of 127 samples from patients with lymph node metastases from University of Szeged (13 from cohort 1) and Lund University cohorts (114 from cohort 2). The protocol for sample collection, transportation, and storage is publicly available[2,3]. Cohort 1 is composed of samples stored as FFPE following surgical removal, while samples from cohort 2[1] were preserved as fresh frozen tissue within 30 minutes after surgery. The complete clinical information can be found in **tables S12 and S13**.

The average age ± standard deviation (range) at the time of the metastasis diagnosis was 60.57 years ± 13.71 (24-88 years). Approximately 63% of patients enrolled in the study were males (80 males vs. 47 females). For 83 patients the BRAF mutation status was measured, 45 of them were V600E mutated, 33 wild type, 4 V600K and 1 V600A mutation. Out of the total 101 patients, follow-up clinical data was available. Among them, 63 individuals developed distant metastases, whereas 38 individuals did not. Informed consent was signed by all patients. GDPR was strictly applied and kept. **Table 1** summarizes an overview of the distribution of some of the clinical parameters within the context of BRAF status and progression to distant metastasis groups in the two cohorts under examination. We show how the groups analyzed are balanced regarding some of the most important clinical information. To corroborate our analyses, we used the TCGA repositories for malignant melanoma. The clinical information for the cohort used is described in **table S4.**

**Table 1: Patients’ clinical information.** Clinical information evaluation of the two cohorts used for the analysis of BRAF mutation status and progression towards distant metastasis.

|  | BRAF mutational status analysis | | | | | |
| --- | --- | --- | --- | --- | --- | --- |
|  | **Cohort 1** | | | **Cohort 2** | | |
| Characteristics | BRAF V600E (n=6) | BRAF WT (n=5) | P-value* | BRAF V600E  (n=39) | BRAF WT  (n=28) | P-value* |
| Gender (%) |  |  |  |  |  |  |
| Male | 4 (66.7) | 4 (80) | >0.9999 | 26 (66.67) | 19 (67.86) | >0.9999 |
| Female | 2 (33.33) | 1 (20) |  | 13 (33.33) | 9 (32.14) |  |
|  |  |  |  |  |  |  |
| Median Age (IQR) | 66 (38-71) | 60.5 (50.5-62) | 0.632 | 58 (45-68) | 60 (55.5-71-5) | 0.2803 |
|  |  |  |  |  |  |  |
| Stage (%) |  |  |  |  |  | >0.9999 |
| I | - | 1 (20) | - | - | - |  |
| II | 6 (100%) | 2 (40) |  | - | - |  |
| III | - | - |  | 29 (74.36) | 21 (75) |  |
| IV | - | 2 (40) |  | 10 (25.64) | 7 (25) |  |
| Unknown |  |  |  |  |  |  |
|  |  |  |  |  |  |  |
| Median Tumor content (IQR) | 9.315 (1.21-69.32) | 62.50 (53.57-73.21 | 0.1255 | 82.5 (50-94.75) | 78.07 (55.13-89.04) | 0.3916 |
|  | **Disease progression towards distant metastases** | | | | | |
|  | **Cohort 1** | | | **Cohort 2** | | |
| Characteristics | Yes (n=9) | No (n=4) | P-value* | Yes (n=54) | No (n=34) | P-value* |
| Gender (%) |  |  |  |  |  |  |
| Male | 6 (66.67) | 4 (100) | - | 37 (68.53) | 16 (47.06) | 0.0728 |
| Female | 3 (33.33) | - |  | 17 (31.48) | 18 (52.94) |  |
|  |  |  |  |  |  |  |
| Median Age (IQR) | 60 (39-63) | 65.5 (62-66) | 0.0797 | 63.50 (49.5-71) | 63 (52.25-73) | 0.927 |
|  |  |  |  |  |  |  |
| BRAF mut. (%) |  |  |  |  |  |  |
| BRAF V600K | 2 (22.22) | - | - | 1 (1.85) | 1 (2.94) | - |
| BRAF V600A | - | - |  | 0 | 1 (2.94) |  |
| BRAF V600E | 4 (44.44) | 2 (50) |  | 19 (35.19) | 10 (29.41) |  |
| BRAF WT | 3 (33.33) | 2 (50) |  | 12 (22.22) | 9 (26.47) |  |
| NA | - | - |  | 22 (40.74) | 13 (38.24) |  |
|  |  |  |  |  |  |  |
| Median Tumor content (IQR) | 21.32 (6.06-65.73) | 31.65 (1.24-68.53) | 0.8252 | 83 (49.06-90.88) | 79.38 (55.38-91.19) | 0.7801 |
| *Mann Whitney test, Fisher’s exact test | | | | | | |

***Sample processing and LC-MS/MS analysis***

In brief, the FFPE samples from the Hungary cohort were deparaffinized using EnVision Flex Target Retrieval Solution High pH (Agilent DAKO)[1]. Cell lysis and protein extraction were carried out using the buffer 100 mM TEAB containing 25 mM DTT and 10w/v% SDS, 1 hour incubation at 99 C, and sonication cycles with the Bioruptor plus UCD-300 (Diagenode). Protein digestion was carried out using S-trap technology in accordance with a previously described procedure[1,4]. Fresh frozen tissue from the second cohort was subjected to protein extraction with urea buffer and sonication cycles with the Bioruptor plus UCD-300 (Diagenode). After digestion with Lys-c and Trypsin, the peptides were labeled with TMT 11 plex and fractionated using basic pH reversed-phase liquid chromatography. Labeled and unlabelled peptides were analyzed by nano liquid chromatography (Ultimate 3000 nLC; Thermo Scientific, San José, CA, USA, Bremen Germany) coupled to high-resolution mass spectrometry (Q Exactive HF-X mass spectrometer; Thermo Scientific). Specific details are described in Betancourt et al (2021)[2].

***Bioinformatics analysis***

The proteomics data was normalized by log2 transformation and then standardized by subtracting the median using Perseus Software. Proteome differences between groups were submitted to functional annotation enrichment analyses using the functional annotation enrichment analysis provided by Perseus software v 1.6.15.0. The significance threshold for enriched Pathway annotations was indicated in each case. Significantly dysregulated proteins or transcripts (from The Cancer Genome Atlas (TCGA) repositories) between groups were determined by applying unpaired t-test analysis (p-value < 0.05). They were submitted to STRING platform for functional annotation enrichment and protein-protein relationship network (https://string-db.org) (FDR < 0.05). Cytoscape software v 3.9.1 was used for visualization and clustering of the protein networks aided by MCODE plugging with a degree cutoff = 2, node score cutoff = 0.2, and K-Core = 2. Clusters with scores >4 were selected.

Hierarchical clustering and heatmap visualization of mitochondrial ribosomal proteins were performed in R using the ComplexHeatmap package. For this analysis, we used the data from samples with tumor content higher than 50%. Mann Whitney test (p-value < 0.05) was used to assess the connection between clinical parameters and clusters based on mitochondrial ribosome proteins (MRPs) abundance. Pearson correlation analysis was performed in R studio (FDR<0.05) to look for proteins and pathways associated with variations at MRP levels. Gene Set Enrichment Analysis (GSEA) was used to identify functional molecular signatures associated with the levels of MRPs utilizing hierarchical clustering. Default parameters were applied employing gene set permutation type. Significant biological processes were determined using a FDR threshold of less than 0.25.

***TCGA pathway analysis***

We utilized public data from The Cancer Genome Atlas (TCGA) to further validate our main findings. Survival data for patients with melanoma stage III - IV were obtained from the clinical data resource (TCGA-CDR)[5]. Validated melanoma-specific survival (MSS) was used as the event time from the date of initial diagnosis until the date of death from melanoma, while the censored time was from the date of initial diagnosis until the date of last follow-up or until the date of death from another cause[5,6]. To focus on protein change in the setting of multiple regulatory pathways, we used pathways previously identified by the PARADIGM algorithm[7,8]. The methodology combines gene-expression, copy-number alteration, and interaction data from several databases to identify “SuperPathways.” Each patient had a Z-transformed single-sample gene-set enrichment (ssGSEA) score. Patients without progression free interval (PFI) or pathway data were excluded. We only included mitochondrial-related pathways (13 total) based on literature and pathway names. The association of each mitochondrial pathway with PFI was determined using Cox proportional hazard models with age, sex, and AJCC stage as covariates. Benjamini-Hochberg method was used for multiple testing correction with a FDR of 0.1 (**Table 2**).

**Table 2.** Mitochondrial pathways associated with worse Melanoma Specific Survival (MSS) after multiple-hypothesis correction using Cox regression models. TCGA cohort.

| Pathway | HR (95% CI)^1^ | p-value |
| --- | --- | --- |
| Mitochondrial Iron Sulfur Cluster Biogenesis | 1.50 (1.15 to 1.97) | 0.003 |
| Mitochondrial Protein Import | 1.67 (1.23 to 2.27) | 0.001 |
| Activation, myristolyation of BID and translocation to mitochondria | 0.72 (0.56 to 0.94) | 0.015 |
| Activation of PUMA and translocation to mitochondria | 1.51 (1.05 to 2.17) | 0.025 |
| Vpr mediated induction of apoptosis by mitochondrial outer membrane permeabilization | 1.46 (1.12 to 1.92) | 0.006 |
| Import of palmitoyl CoA into the mitochondrial matrix | 1.65 (1.17 to 2.33) | 0.005 |
| ^1^HR = Hazard Ratio, CI = Confidence Interval |  |  |

**References**

[1] Szadai, L. et al. (2021). Deep Proteomic Analysis on Biobanked Paraffine-Archived Melanoma with Prognostic/Predictive Biomarker Read-Out. *Cancers*. DOI: 10.3390/cancers13236105.

[2] Betancourt, L.H. et al. (2021). The Human Melanoma Proteome Atlas—Complementing the melanoma transcriptome. *Clinical and Translational Medicine*. DOI: 10.1002/ctm2.451.

[3] Betancourt, L.H. et al. (2021). The human melanoma proteome atlas—Defining the molecular pathology. *Clinical and Translational Medicine*. DOI: 10.1002/ctm2.473.

[4] Kuras, M. et al. (2021). Proteomic Workflows for High-Quality Quantitative Proteome and Post-Translational Modification Analysis of Clinically Relevant Samples from Formalin-Fixed Paraffin-Embedded Archives. *Journal of Proteome Research*. DOI: 10.1021/acs.jproteome.0c00850.

[5] Liu, J. et al. (2018). An Integrated TCGA Pan-Cancer Clinical Data Resource to Drive High-Quality Survival Outcome Analytics. *Cell*. DOI: 10.1016/J.CELL.2018.02.052.

[6] Hudis, C.A. et al. (2007). Proposal for standardized definitions for efficacy end points in adjuvant breast cancer trials: The STEEP system. *Journal of Clinical Oncology*. DOI: 10.1200/JCO.2006.10.3523.

[7] Vaske, C.J. et al. (2010). Inference of patient-specific pathway activities from multi-dimensional cancer genomics data using PARADIGM. *Bioinformatics*. DOI: 10.1093/bioinformatics/btq182.

[8] Hoadley, K.A. et al. (2018). Cell-of-Origin Patterns Dominate the Molecular Classification of 10,000 Tumors from 33 Types of Cancer. *Cell*. DOI: 10.1016/J.CELL.2018.03.022.
